# Metabolic phenotypes and risk of end-stage kidney disease in patients with type 2 diabetes

**DOI:** 10.3389/fendo.2023.1103251

**Published:** 2023-05-10

**Authors:** Lijun Zhao, Yutong Zou, Yucheng Wu, Linli Cai, Yuancheng Zhao, Yiting Wang, Xiang Xiao, Qing Yang, Jia Yang, Honghong Ren, Nanwei Tong, Fang Liu

**Affiliations:** ^1^ Department of General Practice Ward/International Medical Center Ward, General Practice Medical Center, West China Hospital of Sichuan University, Chengdu, Sichuan, China; ^2^ Department of Nephrology, Laboratory of Diabetic Kidney Disease, Centre of Diabetes and Metabolism Research, West China Hospital of Sichuan University, Chengdu, Sichuan, China; ^3^ Division of Endocrinology, West China Hospital of Sichuan University, Chengdu, Sichuan, China

**Keywords:** diabetic kidney disease, type 2 diabetes, metabolic phenotype, end-stage kidney disease, prognostic factor

## Abstract

**Background:**

Obesity often initiates or coexists with metabolic abnormalities. This study aimed to investigate the pathological characteristics and the independent or mutual relations of obesity and metabolic abnormalities with end-stage kidney disease (ESKD) in patients with type 2 diabetes (T2D) and associated diabetic kidney disease (DKD).

**Methods:**

A total of 495 Chinese patients with T2D and biopsy-confirmed DKD between 2003 and 2020 were enrolled in this retrospective study. The metabolic phenotypes were based on the body weight index (BMI)-based categories (obesity, BMI ≥ 25.0 kg/m^2^) and metabolic status (metabolically unhealthy status, ≥ 1 criterion National Cholesterol Education Program Adult Treatment Panel III (NCEP/ATP III) excluding waist circumference and hyperglycemia) and were categorized into four types: metabolically healthy non-obesity (MHNO), metabolically healthy obesity (MHO), metabolically unhealthy non-obesity (MUNO), and metabolically unhealthy obesity (MUO). The pathological findings were defined by the Renal Pathology Society classification. Cox proportional hazards models were used to estimate hazard ratios (HRs) for ESKD.

**Results:**

There are 56 (11.3%) MHNO patients, 28 (5.7%) MHO patients, 176 (35.6%) MUNO patients, and 235 (47.5%) MUO patients. The high prevalence of the Kimmelstiel–Wilson nodule and severe mesangial expansion were associated with obesity, whereas severe IFTA was related to metabolically unhealthy status. In the multivariate analysis, the adjusted HR (aHR) was 2.09 [95% confidence interval (CI) 0.99–4.88] in the MHO group, 2.16 (95% CI 1.20–3.88) in the MUNO group, and 2.31 (95% CI 1.27–4.20) in the MUO group compared with the MHNO group. Furthermore, the presence of obesity was insignificantly associated with ESKD compared with non-obese patients (aHR 1.22, 95% CI 0.88–1.68), while the metabolically unhealthy status was significantly associated with ESKD compared to the metabolically healthy status in the multivariate analysis (aHR 1.69, 95% CI 1.10–2.60).

**Conclusion:**

Obesity itself was insignificantly associated with ESKD; however, adding a metabolically unhealthy status to obesity increased the risk for progression to ESKD in T2D and biopsy-proven DKD.

## 1 Introduction

Diabetes has emerged as one of the most severe non-communicable diseases, affecting approximately 536.6 million adults worldwide according to the 10th edition of the International Diabetes Federation (IDF) Diabetes Atlas (1). China ranks first in diabetes prevalence, with estimates of 140 million affected individuals in 2021 (1). Decreasing mortality among those with diabetes is accomplished by improving medical care; however, the prevalence of developing diabetic kidney disease (DKD) has been estimated at approximately 25%–40% (2). DKD has become the leading cause of end-stage kidney disease (ESKD) in China and worldwide. Delaying the development and progression of DKD remains one of the most important fields in China.

The risk of developing and progressing to DKD is associated with genetic, environmental, and lifestyle factors. These include age, sex, race, as well as modifiable risk factors such as obesity. Several studies have shown that abdominal obesity increases the risk of the progression of chronic kidney disease (CKD) (3, 4). Metabolic syndrome, characterized by a cluster of metabolic abnormalities, has also been identified as an independent risk factor for ESKD (5). Obesity often occurs with metabolic disorders, including hyperglycemia, dyslipidemia, and elevated blood pressure. The role of obesity in renal insufficiency is controversial (6–8), and partly depends on the clustering of metabolic abnormality factors. Obesity and metabolic status are categorized into four types of metabolic phenotypes. Metabolically unhealthy was based on the presence of metabolic syndrome and encompasses three of the five components outlined by the criteria from the National Cholesterol Education Program (NCEP) Adult Treatment Panel-III (ATP-III) (9). Previous studies have shown that the prevalence of CKD slowly increased in metabolically healthy non-obesity (MHNO), metabolically healthy obesity (MHO), metabolically unhealthy non-obesity (MUNO), and metabolically unhealthy obesity (MUO) subgroups within a Taiwanese population (10). Several studies have also reported that, compared to the MHNO group, the MHO, MUNO, and MUO groups progressively increased the CKD risk (11, 12). However, the effect of combined effects of the obesity based on body mass index (BMI) and metabolic status on the risk of future ESKD is lacking. Moreover, stratification by obesity and metabolic status management would have a strong impact on both the individual (stigmatization, self-esteem) and society (attention by healthcare professionals or politicians) (13). Thus, it is important to determine whether obesity itself, metabolic abnormalities, or both conditions contribute to the increased risk of ESKD.

Obesity-related glomerulopathy is characterized by glomerulomegaly as well as disorders of podocytes in the presence of focal and segmental glomerulosclerosis lesions with the underlying mechanisms of insulin resistance and blood pressure salt sensitivity (14). While the metabolic syndrome-induced renal injury mechanism is complex, a high prevalence of microvascular disease was observed in patients with metabolic syndrome. The renal pathological findings in patients with T2D and different metabolic phenotypes have not been well described. Based on the above, this study was used to determine the effect of metabolic phenotype on the risk of ESKD and illustrated the renal pathological characteristics of different metabolic phenotypes in patients with T2D and biopsy-proven DKD.

## 2 Materials and methods

### 2.1 Study design and patient selection

**Figure 1:**
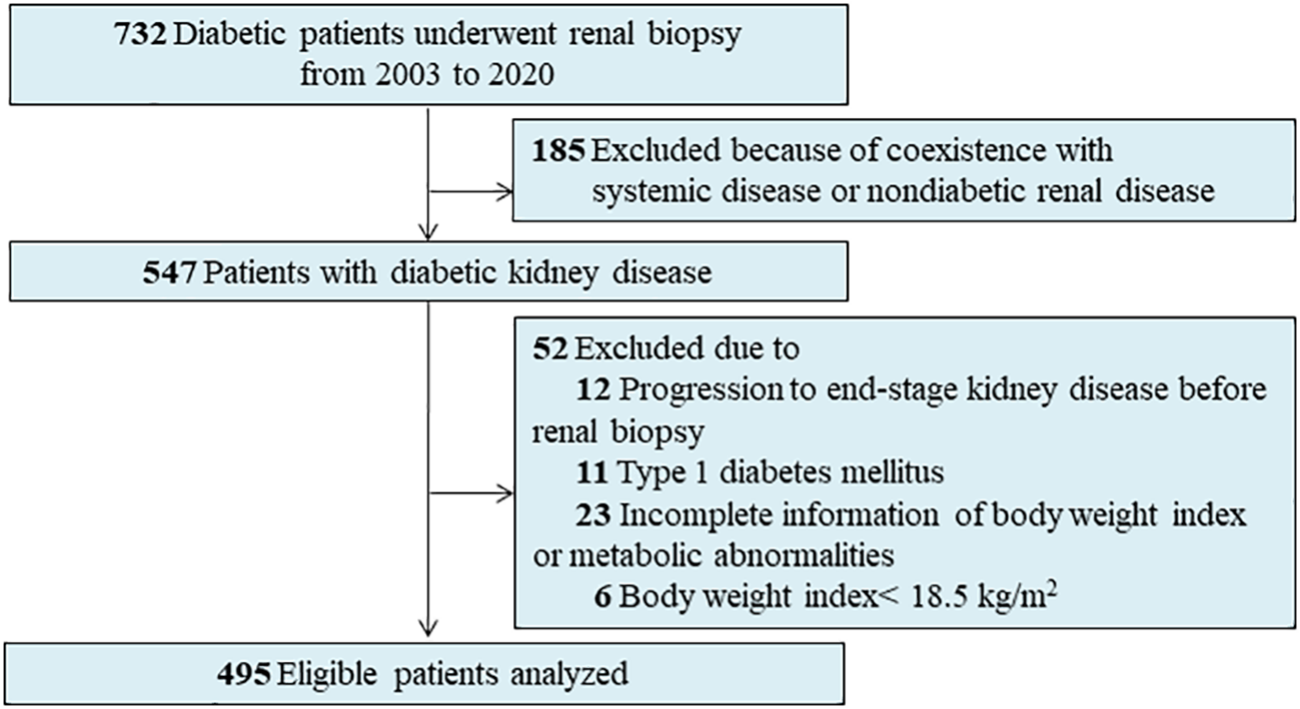
Flowchart of patients in this study.

A total of 732 patients with diabetes who underwent percutaneous renal biopsy between 2003 and 2020 at the West China Hospital of Sichuan University were reviewed. Indications for renal biopsy include diabetes and renal damage with persistent albuminuria or renal dysfunction, sudden onset overt proteinuria, or hematuria (15). We excluded patients with the following conditions at enrollment: type 1 diabetes (n = 11), missing information on BMI or metabolic abnormalities (n = 23), those with a BMI <18.5 kg/m^2^ (n = 6), progression to ESKD at the time of renal biopsy (n = 12), and co-existing non-diabetic renal disease, like membranous nephropathy or immunoglobulin A nephropathy (IgAN) (n = 185) (**Figure 1**). Finally, 495 adult patients with T2D and biopsy-confirmed DKD were included in the present study. All patients provided informed consent. The study was approved by the Institutional Review Board at the West China Hospital of Sichuan University. This study also complied with the 1964 Helsinki Declaration and its later amendments or comparable ethical standards.

### 2.2 Metabolic phenotype

According to the previous studies (16, 17), we defined the metabolic status using the ATP-III metabolic syndrome definition, excluding the waist circumference ≥80 cm component due to its significant collinearity with BMI. As all the patients in our study had T2D, patients with one or more of the three following components were classified as metabolically unhealthy (18): triglyceride level ≥1.7 mmol/L; high-density lipoprotein (HDL) cholesterol level <1.0 mmol/L in men or <1.3 mmol/L in women; or lipid-lowering medication use; systolic blood pressure ≥130 mmHg or diastolic pressure ≥85 mmHg; or antihypertensive medication use. Patients taking lipid-lowering agents with fibrates and/or statins were recorded as fulfilling the lipid criterion, while patients using antihypertensive drugs were recorded as satisfying the blood pressure criterion. Based on the Asian criteria, the general obesity status was assessed by BMI. Obesity was defined as a BMI of ≥ 25.0 kg/m^2^ (19, 20). Patients with a BMI of 18.5–<23.0 were considered normal weight; for those with a BMI of 23.0–<25.0 were overweight. The patients with normal weight and those who were overweight were collapsed into the non-obesity group. Hence, we categorized four metabolic phenotypes based on the BMI-based categories (non-obesity, obesity) and metabolic status (metabolically healthy status or metabolically unhealthy status): MHNO, MHO, MUNO, and MUO (12).

### 2.3 Clinical and pathological covariates

Baseline demographics and clinical information at the time of the renal biopsy were extracted from the hospital’s electronic medical records system. The estimated glomerular filtration rate (eGFR) was evaluated using the Chronic Kidney Disease Epidemiology Collaboration formula (21). The use of renin–angiotensin–aldosterone system inhibitors, new classes of glucose-lowering agents such as glucagon-like peptide-1 receptor agonists (GLP-1RA), dipeptidyl peptidase-4 (DDP-4) inhibitors, and sodium–glucose cotransporter 2 (SGLT2) inhibitors by the patient for more than half of the follow-up period was defined as treatment. Patients attend follow-up appointments two to four times annually, depending on their clinical condition (15, 22, 23).

Renal biopsy tissues were routinely prepared for light microscopy, immunofluorescence, and electron microscopy using standard procedures. The original immunofluorescence, microscopic, and electron microscopy images were used to confirm a diagnosis of DKD according to the basis of the Renal Pathology Society (RPS) classification (24). The glomerular classifications were categorized into five classes, which were class I, class IIa, class IIb, class III, and class IV. Interstitial fibrosis and tubular atrophy (IFTA) and interstitial inflammation were scored from 0 to 2. Arteriolar hyalinosis and arteriosclerosis were assessed and scored according to the RPS-DKD classification (24). In addition to the RPS-DKD classification, other pathological lesions, including global glomerulosclerosis, segmental sclerosis, extracapillary hypercellularity **(**EXHC), the Kimmelstiel–Wilson (KW) lesion, mesangial proliferation, and capillary microaneurysms, were evaluated (25). The detailed definition of pathological parameters is listed in **Supplementary Table 1**.

### 2.4 Renal outcomes

Renal outcome was defined by the progression to ESKD, which was defined as eGFR <15 ml/min/1.73 m^2^ or the need for chronic renal replacement therapy (21). All patients were followed up until January 2022.

### 2.5 Statistical analysis

Continuous datasets were expressed as the mean and standard deviation (SD) if normally distributed, or as the median and interquartile range (IQR) if not normally distributed. Categorical datasets were expressed as counts and percentages. In patients with different types of metabolic phenotypes, differences in the continuous datasets were analyzed using one-way analysis of variance (ANOVA). Categorical datasets were analyzed by the chi-squared test or Fisher’s exact test. Differences in the continuous datasets between patients with or without obesity or metabolically healthy were analyzed by Student’s *t*-test or the Wilcoxon test, while the categorical datasets were analyzed using the chi-squared test or Fisher’s exact test (26).

Survival curves for different metabolic phenotypes were generated using the Kaplan–Meier method and log-rank tests. The Cox proportional hazard model was to explore hazard ratios (HRs) for ESKD. Baseline hemoglobin A1c (HbA1c) levels were missing for eight individuals. Multiple imputation methods were used to derive multivariable models. Three multivariable proportional hazard models, both of which included clinical parameters (age, sex, baseline eGFR, and proteinuria), were used in the present study. Age and sex were chosen based on biological plausibility. The second multivariable model incorporated the above parameters plus smoking status, HbA1c, hemoglobin, serum albumin concentration, usage of renin–angiotensin–aldosterone system inhibitors, and new classes of glucose-lowering agents. Clinical covariates such as HbA1c and serum albumin were selected as potential confounders because of their significance in the univariate analysis or association with ESKD in previous studies (27). The third model was the covariates in model 2 plus pathological parameters. Parameters with *p <*0.05 in the third adjusted model were significant predictors of prognosis.

We performed a series of sensitivity analyses to test the robustness of the results. First, we repeated the main analysis after excluding the first year of follow-up to minimize the potential for reverse causality (28). Second, we reexamined the metabolic phenotypes using the NCEP ATPIII definition of metabolic syndrome (having three of the five criteria) for the determination of metabolically unhealthy status. If the waist circumference was not obtained, the BMI was considered a risk factor for adiposity (29).

All statistical analyses were performed using Stata version 14.0 (Stata Corp. LLC, College Station, TX, USA). Statistical significance was accepted at *p <*0.05.

## 3 Results

### 3.1 Baseline clinical characteristics

**Table 1** shows the baseline clinical characteristics of patients. The mean age of the patients was 51 years, and 346 (69.9%) were men. The median baseline eGFR was 59.7 ml/min/1.73 m^2^, and the median 24-hour proteinuria was 4.20 g/d. The mean BMI was 25 kg/m^2^, and the mean Hb1Ac was 7.6%. A total of 53.1% (263) patients were obese, and 46.9% (232) patients were non-obese. Approximately 17.0% (84) were in a metabolically healthy status, and 83.0% (411) were in a metabolically unhealthy status. Obese patients have a higher prevalence among men, and higher systolic/diastolic blood pressure than non-obese patients. Compared with non-obese patients, obese patients have a higher percentage of Tibetans and higher hemoglobin levels. The eGFR and proteinuria levels showed no significant difference between the non-obese group and the obese group (**Supplementary Table 2**). When patients were stratified by metabolic status, the sex and race distributions were similar between the patients with a metabolically healthy status and those with a metabolically unhealthy status (**Supplementary Table 3**). However, the eGFR level was significantly lower in patients with a metabolically unhealthy status than in those with a metabolically healthy status. Proteinuria showed no difference between the two groups (**Supplementary Table 3**).

**Table 1:** Clinical characteristics of patients with different metabolic phenotypes.

Characteristics	MHNO (n = 56)	MHO (n = 28)	MUNO (n = 176)	MUO (n = 235)	*P*-value
Age, mean (SD), y	51 (10)	51 (8)	51 (9)	51 (10)	0.98
Sex, Male, n (%)	38 (67.9)	24 (85.7)	111 (63.1)	173 (73.6)	0.03
Race, Tibetan, n (%)	6 (10.7)	7 (25.0)	15 (8.5)	34 (14.5)	0.06
Smoking, Never/Ex/Current, (n)	34/5/17	16/3/9	103/25/48	116/42/77	0.405
Body mass index, mean (SD), kg/m^2^	22 (2)	24 (3)	22 (2)	28 (3)	<0.001
Systolic blood pressure, mean (SD), mmHg	121 (9)	122 (9)	146 (24)	148 (23)	<0.001
Diastolic blood pressure, mean (SD), mmHg	78 (6)	79 (6)	85 (14)	88 (13)	<0.001
History of diabetic retinopathy, n (%)	31 (55.4)	11 (39.3)	100 (56.8)	120 (51.1)	0.31
Duration of diabetes, median (IQR), months	96 (36–120)	84 (36–132)	96 (36–144)	96 (40–132)	0.73
Hemoglobin A1c, median (IQR), %	7 (5.9–7.9)	6.9 (5.7–8.4)	7.3 (6.3–8.8)	7.4 (6.3–8.6)	0.29
Hemoglobin A1c, median (IQR), mmol/mol	53 (41–63)	52 (39–68)	56 (45–73)	57 (45–70)	0.29
Hemoglobin, mean (SD), g/L	114 (23)	124 (27)	115 (25)	124 (28)	<0.01
Serum albumin, mean (SD), g/L	33.8 (7.1)	34 (7)	33 (7.5)	34.5 (8)	0.25
Fasting plasma glucose, median (IQR), mg/dL	127 (94.5–190.8)	110.4 (90.6–141.6)	132.6 (101.5–187)	135.7 (104.8–176.4)	0.08
Estimated glomerular filtration rate, median (IQR), ml/min/1.73 m^2^	67.5 (49.1–98.7)	67.3 (52.3–103.7)	58 (42.8–86)	58 (38.5–90.2)	0.04
24-h proteinuria, median (IQR), g/d	3.76 (1.33–5.56)	3.81 (1.75–7.5)	4.59 (2.28–7.04)	4.26 (1.84–7.92)	0.42
Hematuria, n (%)	26 (46.4)	15 (53.6)	78 (44.3)	90 (38.3)	0.30
Uric acid, mean (SD), mg/dl	0.6 (0.1)	0.6 (0.1)	0.6 (0.1)	0.7 (0.2)	0.03
Triglyceride, median (IQR), mg/dl	114.7 (82.4–134.6)	115.2 (84.1–140.4)	160.3 (117.8–224.1)	172.7 (124–263.1)	<0.001
Cholesterol, median (IQR), mg/dl	181.7 (163–210.4)	188.1 (163.8–228.3)	198 (165.7–245.6)	194.5 (160.5–241.7)	0.11
High-density lipoprotein cholesterol, median (IQR), mg/dl	58.4 (50.3–79.5)	51.3 (42.2–63)	50.7 (39.1–69.2)	45.2 (37.1–59.9)	<0.001
Low-density lipoprotein cholesterol, median (IQR), mg/dl	101.5 (72.1–123.5)	113.7 (96.7–143.9)	114.8 (79.7–148.1)	105.2 (76.6–141.9)	0.07
Renin–angiotensin–aldosterone system inhibitors, n (%)	43 (76.8)	22 (78.6)	135 (76.7)	185 (78.7)	0.96
New oral hypoglycemic agents, n (%)	14 (25.0)	10 (35.7)	61 (34.7)	78 (33.2)	0.59
Insulin therapy, n (%)	37 (66.1)	19 (67.9)	121 (68.8)	157 (66.8)	0.95

Data are presented as the mean (standard) for continuous variables with symmetric distribution, median (25th-75th percentiles) for continuous variables with asymmetric distribution, or percentages for categorical variables.

SD, standard deviation; IQR, interquartile range.

When stratified by metabolic phenotypes, there are 56 (11.3%) MHNO patients, 28 (5.7%) MHO patients, 176 (35.6%) MUNO patients, and 235 (47.5%) MUO patients. **Table 1** displays the baseline clinical characteristics among the four groups, with age being similar among all groups. However, patients with MHO and MUO have a higher percentage of males than the MHNO and MUNO groups. The systolic and diastolic blood pressure levels slowly increased in patients with MHNO compared to those with MUO. The median eGFR was 67.5 ml/min/1.73 m^2^ in MHNO, 67.3 ml/min/1.73 m^2^ in MHO, 58.0 ml/min/1.73 m^2^ in MUNO, and 58.0 ml/min/1.73 m^2^ in MUO. The median eGFR decreased slowly from the MHNO group to the MUO group. Of note, the triglyceride concentration slowly increased while the HDL concentration slowly decreased from the MHNO group to the MHO, MUNO, and MUO groups. However, proteinuria, duration of diabetes, HbA1c, and fasting plasma glucose showed no significant difference among the four groups.

### 3.2 Renal pathological changes

**Supplementary Table 4** shows the renal pathological changes in obese patients and non-obese patients. The KW nodule is a typical renal structural change in DKD. The results showed that obese patients have severe mesangial expansion and a higher prevalence of KW nodules than non-obese patients. However, RPS glomerular classification, percentages of global glomerulosclerosis or segmental sclerosis, presence of EXHC, microaneurysm, and IFTA score showed no differences between the two groups.

**Supplementary Table 5** shows renal pathological changes in patients with a metabolically unhealthy and a metabolically healthy status. Patients with a metabolically unhealthy status have higher IFTA, arteriosclerosis, and arteriolar hyalinosis scores compared to those with a metabolically healthy status. However, none of the glomerular lesions, such as mesangial expansion, KW nodule, and EXHC, showed any significant difference between patients with metabolically unhealthy status and those with metabolically healthy status.

Table 2 shows renal pathological findings among the MHNO, MHO, MUNO, and MUO groups. The results demonstrated that patients with MUO have the highest prevalence of KW nodules among the four groups. Compared with the MHNO group, mesangial expansion was much more severe in patients in the MHO, MUNO, and MUO groups. Furthermore, the prevalence of severe IFTA (scores 2 and 3) was significantly higher in the MUNO and MUO groups compared to the MHNO and MHO groups. However, interstitial inflammation, arteriosclerosis score, or arteriolar hyalinosis scores showed no significant difference among the four groups (**Table 2**).

**Table 2:** Renal pathological characteristics of patients with and without metabolic phenotype.

Characteristics	MHNO (n = 56)	MHO (n = 28)	MUNO (n = 176)	MUO (n = 235)	*P*-value
Renal Pathology Society classification^†^, n (%)					0.26
I + IIa	15 (26.8)	8 (28.6)	43 (24.4)	54 (23.0)	
IIb	16 (28.6)	7 (25.0)	36 (20.5)	35 (14.9)	
III	20 (35.7)	11 (39.3)	73 (41.5)	103 (43.8)	
IV	5 (8.9)	2 (7.1)	24 (13.6)	43 (18.3)	
Global glomerulosclerosis, (%)	25.9 (10–38.5)	14.3 (9.2–39.2)	25 (11.8–47.1)	29.4 (14.3–50)	0.91
Segmental sclerosis, (%)	0 (0–6.9)	0 (0–9.6)	0 (0–11.1)	0 (0–10.8)	0.93
Presence of Kimmelstiel–Wilson nodule, n (%)	20 (35.7)	13 (46.4)	74 (42.0)	134 (57.0)	<0.01
Presence of extracapillary hypercellularity, n (%)	6 (10.7)	1 (3.6)	15 (8.5)	15 (6.4)	0.54
Presence of microaneurysm, n (%)	27 (48.2)	6 (21.4)	81 (46.0)	101 (43.0)	0.09
Mesangial expansion					<0.001
score 0	3 (5.4)	0 (.0)	4 (2.3)	6 (2.6)	
score 1	36 (64.3)	4 (14.3)	80 (45.5)	87 (37.0)	
score 2	17 (30.4)	24 (85.7)	92 (52.3)	142 (60.4)	
Interstitial fibrosis and tubular atrophy^†^, n (%)					0.01
score 0	1 (1.8)	4 (14.3)	6 (3.4)	10 (4.3)	
score 1	34 (60.7)	17 (60.7)	93 (52.8)	100 (42.6)	
score 2	17 (30.4)	6 (21.4)	52 (29.5)	93 (39.6)	
score 3	4 (7.1)	1 (3.6)	25 (14.2)	32 (13.6)	
Interstitial inflammation^†^, n (%)					0.16
score 0	1 (1.8)	3 (10.7)	5 (2.8)	8 (3.4)	
score 1	44 (78.6)	16 (57.1)	119 (67.6)	172 (73.2)	
score 2	11 (19.6)	9 (32.1)	52 (29.5)	55 (23.4)	
Arteriosclerosis^†^, n (%)					0.16
score 0	9 (16.1)	5 (17.9)	18 (10.2)	30 (12.8)	
score 1	32 (57.1)	15 (53.6)	77 (43.8)	104 (44.3)	
score 2	15 (26.8)	8 (28.6)	81 (46.0)	101 (43.0)	
Arteriolar hyalinosis^†^, n (%)					0.09
score 0	9 (16.1)	7 (25.0)	18 (10.2)	27 (11.5)	
score 1	21 (37.5)	9 (32.1)	46 (26.1)	63 (26.8)	
score 2	26 (46.4)	12 (42.9)	112 (63.6)	145 (61.7)	

Data are presented as percent for categorical variables. ^†^ Defined by Renal Pathology Society Diabetic Kidney Disease Classification.

### 3.3 Metabolic phenotype and risk of ESKD

**Figure 2:**
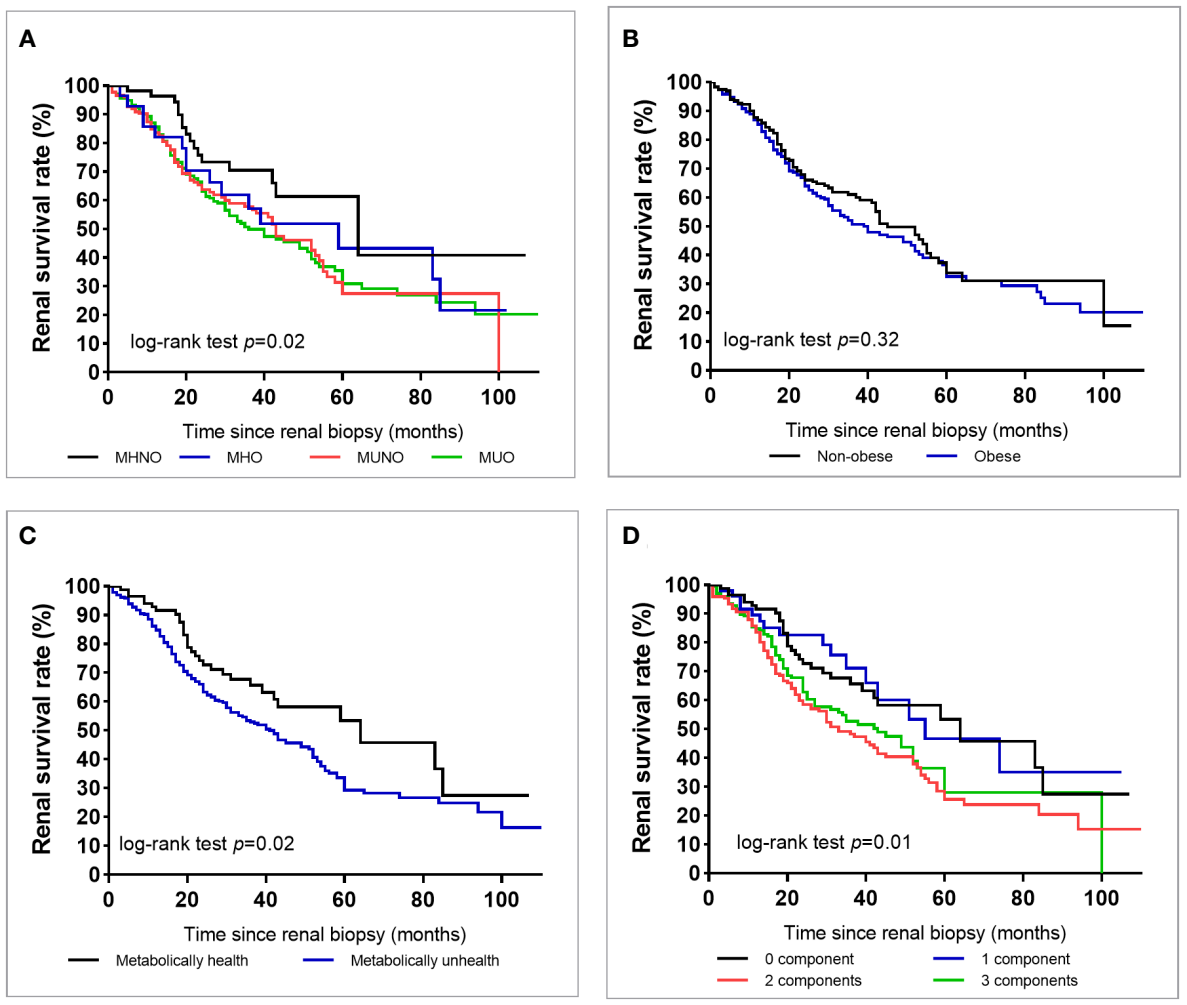
Survival rate of end-stage kidney disease in all patients with type 2 diabetes according to the metabolic phenotype **(A)**, obesity or not **(B)**, metabolically healthy status **(C)**, or according to the number of metabolic abnormalities except for diabetes **(D)**.

During the median follow-up of 29 months, a total of 231 (46.7%) patients reached ESKD, and **Figure 2A** shows that the survival rate for renal outcome decreased from the MHNO group to the MUO group. **Figure 2B** shows that the renal survival rate was similar between non-obese and obese patients. When stratified by metabolic status, patients who were metabolically unhealthy demonstrated a lower renal survival rate than those who were metabolically healthy (**Figure 2C**). An increase in the number of cardiometabolic risk components was associated with an increased incidence of progression to ESKD (log-rank test, *p* = 0.01) (**Figure 2D**).

**Table 3** shows the crude and multivariate-adjusted HRs for progression to ESKD based on the baseline cohort, using the MHNO group as the reference category. Univariate analysis showed that, compared with MHNO, the HR of progression to ESKD in diabetic patients with MHO, MUNO, and MUO was 1.60, 95% confidence interval [CI] 0.99–3.25, 1.92, 95% CI 1.12–3.29, and 1.95, 95% CI 1.16–3.28, respectively. The crude HR of HbA1c and diabetes duration for the progression to ESKD was 0.93 (95% CI 0.86–1.01) and 1.01 (95% CI 0.99–1.01) in patients with T2D, respectively. In the multivariate analysis, the adjusted HRs were 2.09 (95% CI 0.99–4.88) in the MHO, 2.16 (95% CI 1.20–3.88) in the MUNO, and 2.31 (95% CI 1.27–4.20) in the MUO groups compared with the MHNO group used as a reference. Additionally, in the multivariate analysis, HbA1c was not associated with future ESKD (HR 1.05, 95% CI 0.97–1.12).

**Table 3:** Univariate and multivariate analyses for ESKD in patients with type 2 diabetes and diabetic kidney disease.

Variables	Univariate Models hazard ratio (95% confidence interval)	*p*-value	Multivariable model 1^a^ adjusted hazard ratio (95% confidence interval)	*p*-value	Multivariable model 2^b^ adjusted hazard ratio (95% confidence interval)	*p*-value	Multivariable model 3^c^ adjusted hazard ratio (95% confidence interval)	*p* value
Metabolic phenotype								
MHNO	1 (reference)		1 (reference)		1 (reference)		1 (reference)	
MHO	1.60 (0.99–3.25)	0.05	1.86 (0.86–4.03)	0.12	3.17 (1.44–6.97)	<0.01	2.09 (0.99–4.88)	0.06
MUNO	1.92 (1.12–3.29)	0.02	1.76 (1.00–3.08)	0.05	2.15 (1.22–3.79)	0.01	2.16 (1.2–3.88)	0.01
MUO	1.95 (1.16–3.28)	0.01	1.86 (1.08–3.2)	0.03	2.86 (1.62–5.06)	<0.001	2.31 (1.27–4.2)	0.01
Obesity	1.14 (0.88–1.48)	0.33	1.19 (0.9–1.56)	0.22	1.57 (0.97–2.10)	0.06	1.22 (0.88–1.68)	0.22
Metabolically unhealthy status^†^	1.59 (1.09–2.32)	0.02	1.45 (0.96–2.19)	0.08	1.71 (1.12–2.6)	0.01	1.69 (1.10–2.60)	0.02

a Adjusted for age, sex, baseline estimated glomerular filtration rate, 24-h proteinuria, uric acid, hemoglobin A1c, and serum albumin concentration.

b Adjusted for the parameters in multivariable model a plus hemoglobin A1c, hemoglobin, and serum albumin concentration, usage of renin-angiotensin-aldosterone system inhibitors, new oral hypoglycemic agent, and smoking.

c Adjusted for the parameters in multivariable model b plus pathological parameters (Renal Pathology Society glomerular classifications, interstitial fibrosis and tubular atrophy, interstitial inflammation, arteriosclerosis, arteriolar hyalinosis, Kimmelstiel–Wilson nodule, and mesangial expansion).

^†^Comparison to metabolically healthy status.

To illustrate whether obesity or metabolic status is associated with renal outcome, we conducted Cox proportional HRs stratified by obesity or metabolic status (**Table 3**). The results showed that in the multivariate analysis, the presence of obesity itself was insignificantly associated with ESKD in patients with T2D compared with non-obese patients (aHR 1.22, 95% CI 0.88–1.68). When stratified by metabolic status, the metabolically unhealthy status significantly increased the risk for ESKD compared with the metabolically healthy status (aHR 1.69, 95% CI 1.10–2.60). Moreover, no significant interactions were found between obesity and metabolic status for predicting future ESKD (**Supplementary Table 6**).

### 3.4 Subgroup analysis and sensitivity analysis

In the subgroup analysis, the associations between metabolic phenotypes and renal outcome were slightly stronger among those aged >51 years, male, patients with a baseline HbA1c >7%, or with a baseline eGFR <60 ml/min/1.73 m^2^. The adjusted HR was progressively higher in MHO, MUNO, and MUO as compared to the MHNO phenotype (**Supplementary Figure 1**).

In the sensitivity analyses, the relationships between metabolic phenotypes and renal outcome persisted after excluding the first one-year follow-up, which showed consistent positive associations with MHO, MUNO, MUO, and ESKD when compared to the MHNO group (**Supplementary Table 7**). Furthermore, we defined a metabolically unhealthy status as the presence of metabolic syndrome. We performed sensitivity analyses when using the metabolic syndrome NCEP ATPIII definition and obtained similar results (**Supplementary Table 8**).

## 4 Discussion

In this retrospective cohort of patients with T2D and biopsy-proven DKD, approximately half of the patients were obese and had a metabolically unhealthy status. The adjusted HR was progressively higher in MHO, MUNO, and MUO as compared to the MHNO phenotype. Of note, the presence of obesity was not significantly associated with ESKD compared with non-obesity patients, while a metabolically unhealthy status was significantly associated with ESKD compared with a metabolically healthy status in multivariate analysis. Regarding renal pathological characteristics, the high prevalence of KW nodules and severe mesangial expansion was associated with obesity, whereas severe IFTA was associated with a metabolically unhealthy status.

To our knowledge, this is the first study to investigate the association between metabolic phenotype and renal outcome in biopsy-proven DKD patients. Patients with biopsy-confirmed DKD were recruited to uniformly assess renal pathological changes, while the underlying effect of non-diabetic renal diseases on renal outcome was excluded. The link between progressive kidney disease in obese patients and metabolic abnormalities is of enormous public health importance. The role of obesity in renal insufficiency in diabetes is controversial according to the literature. Chung et al. (7) conducted a longitudinal study on 1,187 diabetic patients in Taiwan, showing that diabetic patients who are obese were more likely to have CKD. However, a prospective 5-year study conducted in the eastern region of Morocco showed that the decline of eGFR in DKD of T2D is not directly influenced by BMI (6). Another large epidemiological study conducted in Manchester, UK, demonstrated that there was no statistically significant difference in the rate of progression of CKD between obese and non-obese T2D patients (30). The results of our study showed that obesity itself was not an independent risk factor for ESKD in T2D. This indicated that obesity increased intraglomerular pressure and played an important role in the incidence of DKD, while it was not an independent risk factor for the progression of DKD in T2D. The major risk factors contributing to eGFR decline remain traditional factors such as a low baseline eGFR. Moreover, our data showed that metabolic abnormalities were significantly associated with the risk of ESKD in T2D after adjusting for traditional factors. Similarly, a cross-sectional study enrolled 14,983 general population participants in Taiwan and reported that a metabolically unhealthy status, but not obesity, was associated with a higher risk of CKD (10). Data from a cohort of 3.5 million individuals recorded by The Health Improvement Network in the United Kingdom showed that although cardiometabolic risk increased from normal weight to overweight and obesity, it was more pronounced with an increasing number of metabolic abnormalities (31). It was suggested that metabolic abnormalities could be the intermediate factors linking obesity to future ESKD. Our results showed that obesity itself was insignificantly associated with ESKD; however, their association was enhanced and became significant when adding metabolic status, supporting this suggestion. Furthermore, a significant relationship was found between metabolically unhealthy status and the risk of ESKD, demonstrating their simultaneous contributions to the disease.

MHO, due to its low prevalence (only 5.7% in our cohort), was often ignored by physicians. MHO is characterized as obesity with a low burden of metabolic abnormality, which has been considered a “benign condition” in the past few years. In different European countries, the prevalence of MHO varied from 2% to 28% (13). Meta-analyses of prospective studies demonstrated that MHO is associated with a significantly lower incidence of T2D (32) and cardiovascular disease (32, 33). However, our results revealed that MHO did not associate with renal outcome, which was consistent with a previous study (34). Despite these, we reveal that in subgroups of ages greater than 51 years old, male, HbA1c >7%, and eGFR <60 ml/min/1.73 m^2^, MHO significantly increased the risk for progression to ESKD compared with MHNO. This result indicates that MOH still needs rapid intervention in this patient group.

MHO, MUNO, and MUO are reported to be transient phenotypes (13). Previous studies reported that the cardiovascular disease risk increased in women who converted from MHO to MUO compared to those with stable MHO, which was primarily driven by the incidence of T2D and hypertension (33). In this study, we found that systolic/diastolic blood pressure was significantly higher in patients with MUO than in those with MHO, which highlights the importance of intensive blood pressure and blood glucose therapy in a pragmatic approach. Compared with MHO, MUO was considered an alteration in the distribution of ectopic fat. The ectopic fat accumulation triggered an inflammatory cascade, and lipotoxicity was considered an inducer of metabolic dysfunction (35). The transitions between MHO and MUO are not unidirectional and may change over time. Patients who underwent obesity surgery or strict weight-loss interventions may lead the MUO phenotype transition to MHO or even the MHNO phenotype (36). A nationwide population-based study reported that patients who converted to MHNO had a 2% decrease in risk, whereas patients who evolved to MUNO or MUO had a 60%–68% increased risk of CKD incidence compared to the stable MHNO group (28). Thus, we performed a sensitivity analysis by excluding the first one-year follow-up to test the robustness of the results, which showed consistent positive associations with MHO, MUNO, MUO, and ESKD. Based on the above results, combination therapy for both diabetes and obesity appear to be a promising tool for the future pharmacotherapy of DKD. Indeed, the first glucagon-like peptide 1 (GLP-1)/glucagon coagonist (cotadutide) improved glycemic control, weight loss, and even improved metabolic profiles in patients who were overweight or obese and had T2D in a Phase 2b clinical trial (37). Another novel dual glucose-dependent insulinotropic polypeptide (GIP) and GLP-1 receptor agonist, tirzepatide, not only reduces body weight but also improves the metabolite profile in patients with T2D (38, 39). The *post-hoc* analysis of the SURPASS-4 study showed that tirzepatide showed a delayed effect on eGFR decline in patients with T2D (40).

The obesity-related glomerulopathy shares several pathophysiologic factors relevant to renal damage in DKD. The present study showed that the presence of KW nodules and a mesangial expansion degree were severe in obese patients compared to non-obese patients. The mechanism may be related to the renal hemodynamic changes due to obesity. Animal models of obesity-related glomerulopathy showed that the glomerular tuft volume increased exponentially in relation to body weight gain (41). The numerical density of podocytes decreases as the glomerular diameter increases under persistent renal hyperfusion, thereby causing the decreased extension of podocytic processes to cover the expanded area. This induced a loss in protein selectivity and triggered matrix deposition (42). However, the pathological findings were much different when stratified by metabolic status. Patients with a metabolically unhealthy status have more severe interstitial and microvascular changes (arteriosclerosis and arteriolar hyalinosis) than those with a metabolically healthy status, which is consistent with previous studies (29, 43). The underlying mechanism of metabolic abnormalities induced renal tubular injury may be associated with oxidative stress and cytochrome-C-induced apoptosis (44). The microvascular changes may be linked to greater blood pressure observed in patients with a metabolically unhealth status (43). Coexisting metabolic abnormalities and renal vascular changes synergistically aggravate tubular mitochondrial damage and dysfunction, ultimately leading to renal structural injury and renal insufficiency (45).

Albuminuria is one of the early warning signs of CKD. A previous study showed a relationship between metabolic syndrome and albuminuria, especially in non-East Asian populations (46). But in patients with renal transplant, albuminuria was more associated with elevated systolic blood pressure and hyperglycemic status than with metabolic syndrome (47). Thus, the absence of an association between proteinuria and unhealthy metabolic status may partly be related to the different ethnicities and advanced DKD in this study.

Univariate and multivariate analysis showed that diabetes duration and baseline HbA1c were not associated with progression to ESKD in patients with T2D (data not shown), which was consistent with previous studies (21, 25, 48). In a prospective analysis of patients with T2D, the coefficient of HbA1c variation, not the mean of serially measured fasting plasma glucose, was predictive of the development of renal complications and diabetes-related outcomes (49). A wide variance of HbA1c reflects a more complicated clinical course, suboptimal use of medications, and/or self-management (49). The absence of an association between diabetes duration and renal outcome may be explained by the inability to establish the time of onset of T2DM with certainty. Many individuals have undiagnosed diabetes and impaired glucose tolerance for extended periods, leading to inaccurate assessments of diabetes duration (50).

There are several limitations to this study. First, due to the retrospective observational design, causal relationships between metabolic phenotype and DKD progression cannot be inferred from the results. A prospective study conducted among patients with T2D will be useful to validate our conclusions. Second, our study consisted of a single-center cohort, thus lacking external validity. However, biochemical measurements using the same standard method improved the reliability of our data. Third, no phenotypic changes were recorded during the follow-up time. A limited patient sample size of individuals with MHO was another limitation of this study. The negative association between MHO and renal outcome in patients with T2D cannot be excluded, with the limited number of patients and low incidence of renal events resulting in insufficient power. However, the absence of an interaction between obesity and a metabolically unhealthy status led to the potentially impactful conclusion that obesity itself was not significantly associated with renal outcome in T2D. A large cohort sample size would be useful to validate our conclusion.

In conclusion, this retrospective cohort study demonstrated that the combination of obesity and metabolic health status was associated with an increased risk of ESKD in T2D and biopsy-proven DKD. The highest risk was observed in participants with both obesity and a metabolically unhealthy status. Specifically, MUNO and MUO were both associated with increased risk of ESKD, suggesting that metabolic profiles need to be regularly monitored to reduce future ESKD risk.

## Data availability statement

The original contributions presented in the study are included in the article/**Supplementary Material.** Further inquiries can be directed to the corresponding author.

## Ethics statement

The studies involving human participants were reviewed and approved by The institutional review board at the West China Hospital of Sichuan University. The patients/participants provided their written informed consent to participate in this study.

## Author contributions

LZ analyzed the data, interpreted the results, and drafted the manuscript. FL analyzed and interpreted data, edited/revised, and approved the final version of the manuscript. YTZ, YCW, HR, YTW, YCZ, XX, JY, QY, and LC carried out the data collecting and recording. NT contributed to the discussion. FL is the guarantor of this work and had full access to all the data in this study and takes responsibility for the integrity of the data. All authors listed have made a substantial, direct, and intellectual contribution to the work and approved it for publication.

## References

[1] Sun H Saeedi P Karuranga S Pinkepank M Ogurtsova K Duncan BB. IDF diabetes atlas: global, regional and country-level diabetes prevalence estimates for 2021 and projections for 2045. Diabetes Res Clin Pract (2022) 183:109119. doi: 10.1016/j.diabres.2021.109119 34879977 PMC11057359

[2] Jung CY Yoo TH. Pathophysiologic mechanisms and potential biomarkers in diabetic kidney disease. Diabetes Metab J (2022) 46(2):181–97. doi: 10.4093/dmj.2021.0329 PMC8987689 35385633

[3] Bae EH Lim SY Jung JH Oh TR Choi HS Kim CS. Obesity, abdominal obesity and chronic kidney disease in young adults: A nationwide population-based cohort study. J Clin Med (2021) 10(5):1065–78. doi: 10.3390/jcm10051065 PMC7962022 33806552

[4] Evangelista LS Cho WK Kim Y. Obesity and chronic kidney disease: a population-based study among south koreans. PloS One (2018) 13(2):e0193559. doi: 10.1371/journal.pone.0193559 29489920 PMC5831002

[5] Lee YB Han K Kim B Jun JE Lee SE Ahn J. Risk of end-stage renal disease from chronic kidney disease defined by decreased glomerular filtration rate in type 1 diabetes: a comparison with type 2 diabetes and the effect of metabolic syndrome. Diabetes Metab Res Rev (2019) 35(8):e3197. doi: 10.1002/dmrr.3197 31222888

[6] Bentata Y Latrech H Abouqal R. Does body mass index influence the decline of glomerular filtration rate in diabetic type 2 patients with diabetic nephropathy in a developing country? Ren Fail (2014) 36(6):838–46. doi: 10.3109/0886022x.2014.899472 24673339

[7] Chung HF Al Mamun A Huang MC Long KZ Huang YF Shin SJ. Obesity, weight change, and chronic kidney disease in patients with type 2 diabetes mellitus: a longitudinal study in Taiwan. J Diabetes (2017) 9(11):983–93. doi: 10.1111/1753-0407.12514 27976508

[8] Shibata I Shibata M Sato KK Takeuchi Y Okamura K Koh H. Body mass index and the risk of persistent proteinuria in middle-aged men: the Kansai healthcare study. Am J Nephrol (2022) 53(2-3):191–8. doi: 10.1159/000521885 35139520

[9] Expert Panel on Detection, Evaluation, and Treatment of High Blood Cholesterol in Adults. Executive summary of the third report of the national cholesterol education program (NCEP) expert panel on detection, evaluation, and treatment of high blood cholesterol in adults (Adult treatment panel III). Jama (2001) 285(19):2486–97. doi: 10.1001/jama.285.19.2486 11368702

[10] Chen HY Lu FH Chang CJ Wang RS Yang YC Chang YF. Metabolic abnormalities, but not obesity per se, associated with chronic kidney disease in a Taiwanese population. Nutr Metab Cardiovasc Dis (2020) 30(3):418–25. doi: 10.1016/j.numecd.2019.09.029 31744713

[11] Alizadeh S Esmaeili H Alizadeh M Daneshzad E Sharifi L Radfar H. Metabolic phenotypes of obese, overweight, and normal weight individuals and risk of chronic kidney disease: a systematic review and meta-analysis. Arch Endocrinol Metab (2019) 63(4):427–37. doi: 10.20945/2359-3997000000149 PMC10528657 31365625

[12] Wang Y Sun B Sheng LT Pan XF Zhou Y Zhu J. Association between weight status, metabolic syndrome, and chronic kidney disease among middle-aged and elderly Chinese. Nutr Metab Cardiovasc Dis (2020) 30(11):2017–26. doi: 10.1016/j.numecd.2020.06.025 32826134

[13] Blüher M. Metabolically healthy obesity. Endocr Rev (2020) 41(3):1–16. doi: 10.1210/endrev/bnaa004 PMC7098708 32128581

[14] Ritz E Koleganova N Piecha G. Is there an obesity-metabolic syndrome related glomerulopathy? Curr Opin Nephrol Hypertens (2011) 20(1):44–9. doi: 10.1097/MNH.0b013e3283414ca1 21088574

[15] Zhao L Zou Y Zhang J Zhang R Ren H Li L. Serum transferrin predicts end-stage renal disease in type 2 diabetes mellitus patients. Int J Med Sci (2020) 17(14):2113–24. doi: 10.7150/ijms.46259 PMC7484672 32922172

[16] Liang X Margolis KL Hendryx M Rohan TE Groessl EJ Thomson CA. Metabolic phenotype and risk of colorectal cancer in normal-weight postmenopausal women. Cancer Epidemiol Biomarkers Prev (2017) 26(2):155–61. doi: 10.1158/1055-9965.Epi-16-0761 PMC5301805 28148595

[17] Schweitzer ML Stengel B Legrand K Briançon S Jacquelinet C Combe C. Obesity phenotype and patient-reported outcomes in moderate and severe chronic kidney disease: a cross-sectional study from the CKD-REIN cohort study. Qual Life Res (2019) 28(7):1873–83. doi: 10.1007/s11136-019-02110-2 30659448

[18] Cho YK Lee J Kim HS Park JY Lee WJ Kim YJ. Metabolic health is a determining factor for incident colorectal cancer in the obese population: a nationwide population-based cohort study. Cancer Med (2021) 10(1):220–9. doi: 10.1002/cam4.3607 PMC7826459 33216467

[19] Chen J Kong X Jia X Li W Wang Z Cui M. Association between metabolic syndrome and chronic kidney disease in a Chinese urban population. Clin Chim Acta (2017) 470:103–8. doi: 10.1016/j.cca.2017.05.012 28501388

[20] Zheng X Peng R Xu H Lin T Qiu S Wei Q. The association between metabolic status and risk of cancer among patients with obesity: metabolically healthy obesity vs. metabolically unhealthy obesity. Front Nutr (2022) 9:783660. doi: 10.3389/fnut.2022.783660 35284439 PMC8914254

[21] Zhao L Ren H Zhang J Cao Y Wang Y Meng D. Diabetic retinopathy, classified using the lesion-aware deep learning system, predicts diabetic end-stage renal disease in Chinese patients. Endocr Pract (2020) 26(4):429–43. doi: 10.4158/EP-2019-0512 31968187

[22] Zhao L Wang X Wang T Fan W Ren H Zhang R. Associations between high-altitude residence and end-stage kidney disease in Chinese patients with type 2 diabetes. High altitude Med Biol (2020) 21(4):396–405. doi: 10.1089/ham.2020.0076 33185478

[23] Zhao L Zhang J Lei S Ren H Zou Y Bai L. Combining glomerular basement membrane and tubular basement membrane assessment improves the prediction of diabetic end-stage renal disease. J Diabetes (2021) 13(7):572–84. doi: 10.1111/1753-0407.13150 33352010 PMC8246816

[24] Tervaert TW Mooyaart AL Amann K Cohen AH Cook HT Drachenberg CB. Pathologic classification of diabetic nephropathy. J Am Soc Nephrol (2010) 21(4):556–63. doi: 10.1681/ASN.2010010010 20167701

[25] Zhao L Liu F Li L Zhang J Wang T Zhang R. Solidified glomerulosclerosis, identified using single glomerular proteomics, predicts end-stage renal disease in Chinese patients with type 2 diabetes. Sci Rep (2021) 11(1):4658. doi: 10.1038/s41598-021-83856-z 33633132 PMC7907371

[26] Zhao L Li L Ren H Zou Y Zhang R Wang S. Association between serum alkaline phosphatase and renal outcome in patients with type 2 diabetes mellitus. Renal Failure (2020) 42(1):818–28. doi: 10.1080/0886022x.2020.1804402 PMC7472471 32781868

[27] Zhang J Zhang R Wang Y Li H Han Q Wu Y. The level of serum albumin is associated with renal prognosis in patients with diabetic nephropathy. J Diabetes Res (2019) 2019:7825804. doi: 10.1155/2019/7825804 30911552 PMC6398001

[28] Cho YK Lee J Kim HS Park JY Lee WJ Kim YJ. Impact of transition in metabolic health and obesity on the incident chronic kidney disease: A nationwide cohort study. J Clin Endocrinol Metab (2020) 105(3):e148–e157. doi: 10.1210/clinem/dgaa033 31967306

[29] Zhao L Zou Y Bai L Zhou L Ren H Wu Y. Prognostic value of metabolic syndrome in renal structural changes in type 2 diabetes. Int Urol Nephrol (2022) 54(8):2005–14. doi: 10.1007/s11255-021-03051-x 35043385

[30] Mohsen A Brown R Hoefield R Kalra PA O'Donoghue D Middleton R. Body mass index has no effect on rate of progression of chronic kidney disease in subjects with type 2 diabetes mellitus. J Nephrol (2012) 25(3):384–93. doi: 10.5301/jn.5000062 22241634

[31] Caleyachetty R Thomas GN Toulis KA Mohammed N Gokhale KM Balachandran K. Metabolically healthy obese and incident cardiovascular disease events among 3.5 million men and women. J Am Coll Cardiol (2017) 70(12):1429–37. doi: 10.1016/j.jacc.2017.07.763 28911506

[32] Bell JA Kivimaki M Hamer M. Metabolically healthy obesity and risk of incident type 2 diabetes: a meta-analysis of prospective cohort studies. Obes Rev (2014) 15(6):504–15. doi: 10.1111/obr.12157 PMC4309497 24661566

[33] Eckel N Li Y Kuxhaus O Stefan N Hu FB Schulze MB. Transition from metabolic healthy to unhealthy phenotypes and association with cardiovascular disease risk across BMI categories in 90 257 women (the nurses' health study): 30 year follow-up from a prospective cohort study. Lancet Diabetes Endocrinol (2018) 6(9):714–24. doi: 10.1016/s2213-8587(18)30137-2 29859908

[34] Uehara S Sato KK Koh H Shibata M Kinuhata S Yamada A. The association between metabolically healthy obesity and the risk of proteinuria: the Kansai healthcare study. J Epidemiol (2018) 28(8):361–6. doi: 10.2188/jea.JE20170082 PMC6048297 29628481

[35] Martin-Piedra L Alcala-Diaz JF Gutierrez-Mariscal FM Arenas de Larriva AP Romero-Cabrera JL Torres-Peña JD. Evolution of metabolic phenotypes of obesity in coronary patients after 5 years of dietary intervention: from the CORDIOPREV study. Nutrients (2021) 13(11):4046–59. doi: 10.3390/nu13114046 PMC8624211 34836298

[36] van Vliet-Ostaptchouk JV Nuotio ML Slagter SN Doiron D Fischer K Foco L. The prevalence of metabolic syndrome and metabolically healthy obesity in Europe: a collaborative analysis of ten large cohort studies. BMC Endocr Disord (2014) 14:9. doi: 10.1186/1472-6823-14-9 24484869 PMC3923238

[37] Nahra R Wang T Gadde KM Oscarsson J Stumvoll M Jermutus L. Effects of cotadutide on metabolic and hepatic parameters in adults with overweight or obesity and type 2 diabetes: a 54-week randomized phase 2b study. Diabetes Care (2021) 44(6):1433–42. doi: 10.2337/dc20-2151 PMC8247525 34016612

[38] Pirro V Roth KD Lin Y Willency JA Milligan PL Wilson JM. Effects of tirzepatide, a dual GIP and GLP-1 RA, on lipid and metabolite profiles in subjects with type 2 diabetes. J Clin Endocrinol Metab (2022) 107(2):363–78. doi: 10.1210/clinem/dgab722 34608929

[39] Thomas MK Nikooienejad A Bray R Cui X Wilson J Duffin K. Dual GIP and GLP-1 receptor agonist tirzepatide improves beta-cell function and insulin sensitivity in type 2 diabetes. J Clin Endocrinol Metab (2021) 106(2):388–96. doi: 10.1210/clinem/dgaa863 PMC7823251 33236115

[40] Heerspink HJL Sattar N Pavo I Haupt A Duffin KL Yang Z. Effects of tirzepatide versus insulin glargine on kidney outcomes in type 2 diabetes in the SURPASS-4 trial: Post-hoc analysis of an open-label, randomised, phase 3 trial. Lancet Diabetes Endocrinol (2022) 10(11):774–85. doi: 10.1016/s2213-8587(22)00243-1 36152639

[41] Guo YP Jiang HK Jiang H Tian HY Li L. Lipoxin A4 may attenuate the progression of obesity-related glomerulopathy by inhibiting NF-κB and ERK/p38 MAPK-dependent inflammation. Life Sci (2018) 198:112–8. doi: 10.1016/j.lfs.2018.02.039 29499280

[42] Wei L Li Y Yu Y Xu M Chen H Li L. Obesity-related glomerulopathy: from mechanism to therapeutic target. Diabetes Metab Syndr Obes (2021) 14:4371–80. doi: 10.2147/dmso.S334199 PMC8560069 34737593

[43] Alexander MP Patel TV Farag YMK Florez A Rennke HG Singh AK. Kidney pathological changes in metabolic syndrome: a cross-sectional study. Am J Kidney Dis Off J Natl Kidney Foundation (2009) 53(5):751–9. doi: 10.1053/j.ajkd.2009.01.255 PMC2765403 19339092

[44] Eirin A Woollard JR Ferguson CM Jordan KL Tang H Textor SC. The metabolic syndrome induces early changes in the swine renal medullary mitochondria. Transl Res (2017) 184:45–56.e9. doi: 10.1016/j.trsl.2017.03.002 28363084 PMC5429873

[45] Nargesi AA Zhang L Tang H Jordan KL Saadiq IM Textor SC. Coexisting renal artery stenosis and metabolic syndrome magnifies mitochondrial damage, aggravating poststenotic kidney injury in pigs. J hypertension (2019) 37(10):2061–73. doi: 10.1097/HJH.0000000000002129 PMC6771269 31465309

[46] Rashidbeygi E Safabakhsh M Delshad Aghdam S Mohammed SH Alizadeh S. Metabolic syndrome and its components are related to a higher risk for albuminuria and proteinuria: evidence from a meta-analysis on 10,603,067 subjects from 57 studies. Diabetes Metab Syndr (2019) 13(1):830–43. doi: 10.1016/j.dsx.2018.12.006 30641817

[47] Sipahioglu MH Unal A Yazgac H Tunca O Arikan T Kocyigit I. Relationships between metabolic syndrome, microalbuminuria, and c-reactive protein in Turkish kidney transplant recipients. Transplant Proc (2015) 47(5):1408–12. doi: 10.1016/j.transproceed.2015.04.037 26093730

[48] Zhuo L Zhang N Zou G Chen D Li W. Clinical characteristics and outcomes of biopsy-proven diabetic nephropathy. Front Med (2017) 11(3):386–92. doi: 10.1007/s11684-017-0574-z 28871455

[49] Luk AO Ma RC Lau ES Yang X Lau WW Yu LW. Risk association of HbA1c variability with chronic kidney disease and cardiovascular disease in type 2 diabetes: prospective analysis of the Hong Kong diabetes registry. Diabetes Metab Res Rev (2013) 29(5):384–90. doi: 10.1002/dmrr.2404 23463747

[50] Zimmet P. The burden of type 2 diabetes: are we doing enough? Diabetes Metab (2003) 29(4 Pt 2):6s9–18. doi: 10.1016/s1262-3636(03)72783-9 PMC7129589 14502096

