# Supplementary material for: Metabolic phenotypes and risk of end-stage kidney disease in patients with type 2 diabetes
Source: Front Endocrinol (Lausanne). 2023 May 10;14:1103251. doi: 10.3389/fendo.2023.1103251 (PMC10206309; doi:10.3389/fendo.2023.1103251)
Supplement: Supplementary file 1 [file DataSheet_1.docx]

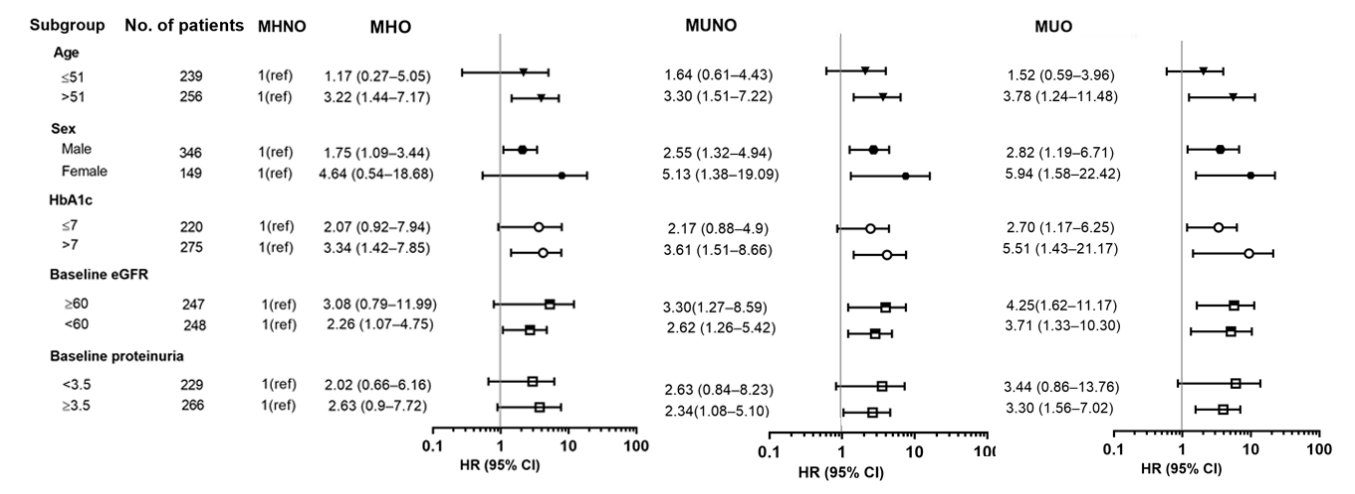


**Supplementary Figure 1. Multivariate analyses for end-stage kidney disease in patients with type 2 diabetes and diabetic kidney disease in the subgroup analysis.**

**Supplementary Table 1. Definition of pathological parameters.**

| Pathological findings | Scores | Definition of score |
| --- | --- | --- |
| **Glomerular lesions** |  |  |
| Renal Pathology Society glomerular class ^a^ |  |  |
|  | I | Mild, nonspecific light microscopy changes with glomerular basement membrane thickening |
|  | IIa | Mild mesangial expansion in ＞25% of mesangium |
|  | IIb | Severe mesangial expansion in ＞25% of mesangium |
|  | III | At least one Kimmelstiel-Wilson nodule |
|  | IV | Advanced diabetic glomerulosclerosis |
| Global glomerulosclerosis |  | (Number of global glomerulosclerosis)/number of all glomeruli (%) |
| Segmental sclerosis |  | (Number of segmental sclerosis)/number of all glomeruli (%) |
| Kimmelstiel-Wilson nodules |  |  |
|  | 0 | Absent |
|  | 1 | Detected one or more lesions in all biopsy specimens |
| Extracapillary hypercellularity |  |  |
|  | 0 | Absent |
|  | 1 | Detected one or more lesions in all biopsy specimens |
| Capillary microaneurysm |  |  |
|  | 0 | Absent |
|  | 1 | Detected one or more lesions in all biopsy specimens |
| Mesangial expansion |  |  |
|  | 1 | Mesangial expansion ＜ Capillary lumen |
|  | 2 | Mesangial expansion = Capillary lumen |
|  | 3 | Mesangial expansion ＞ Capillary lumen |
| **Interstitial lesions** |  |  |
| Interstitial fibrosis and tubular atrophy(IFTA) ^a^ |  |  |
|  | 0 | No IFTA |
|  | 1 | 1%- 25% cortex |
|  | 2 | 25% - 50% cortex |
|  | 3 | ＞50% cortex |
| Interstitial inflammation ^a^ |  |  |
|  | 0 | Absent |
|  | 1 | Infiltration only in relation to IFTA |
|  | 2 | Infiltration in an area without IFTA |
| **Vascular lesions** |  |  |
| Arteriosclerosis ^a^ |  |  |
|  | 0 | No intimal thickening |
|  | 1 | Intimal thickening＜ thickness of media |
|  | 2 | Intimal thickening≥thickness of media |
| Arteriolar hyalinosis ^a^ |  |  |
|  | 0 | Absent |
|  | 1 | At least one area of arteriolar hyalinosis |
|  | 2 | More than one area of arteriolar hyalinosis |

^a^ Defined by Renal Pathology Society Diabetic Kidney Disease Classification. For Cox proportional hazard analyses, class I and class IIa were collapsed together as the reference group for Renal Pathology Society glomerular class.

**Supplementary Table 2. Clinical characteristics of patients with non-obese or obese**

| Characteristics | Non-obesity (n=232） | Obesity (n=263) | *p* value |
| --- | --- | --- | --- |
| Age, mean (SD), y | 51 (9) | 51 (10) | 0.78 |
| Sex, Male, n (%) | 149 (64.2) | 197 (74.9) | 0.01 |
| Race, Tibetan, n(%) | 21 (9.1) | 41 (15.6) | 0.03 |
| Smoking, Never/Ex/Current, (n) | 137/30/65 | 132/45/86 | 0.13 |
| Body mass index, mean (SD), kg/m2 | 22 (2) | 27 (3) | <0.001 |
| Systolic blood pressure, mean (SD), mmHg | 140 (24) | 146 (23) | <0.001 |
| Diastolic blood pressure, mean (SD), mmHg | 83 (13) | 87 (13) | <0.001 |
| History of diabetic retinopathy, n (%) | 131 (56.5) | 131 (49.8) | 0.14 |
| Duration of diabetes, median (IQR), months | 96 (36–132) | 96 (36–132) | 0.77 |
| Hemoglobin A1c, median (IQR), % | 7.1 (6.3–8.6) | 7.4 (6.3–8.6) | 0.83 |
| Hemoglobin A1c, median (IQR), mmol/mol | 54 (45–70) | 57 (45–70) | 0.83 |
| Hemoglobin, mean (SD), g/L | 114.3 (24.4) | 123.6 (28.1) | <0.001 |
| Serum albumin, mean (SD), g/L | 33.2 (7.4) | 34.5 (7.9) | 0.06 |
| Fasting plasma glucose, median (IQR), mg/dL | 129.3 (98.6–187) | 132.3 (102.6–170.3) | 0.74 |
| Estimated glomerular filtration rate, median (IQR), mL/min/1.73 m2 | 59.7 (43.9–89.6) | 60 (39.9–90.4) | 0.59 |
| 24-h proteinuria, median (IQR), g/d | 4.3 (2.24–6.82) | 4.2 (1.84–7.79) | 0.88 |
| Hematuria, n (%) | 104 (44.8) | 105 (39.9) |  |
| Uric acid, mean (SD), mg/dL | 0.6 (0.1) | 0.7 (0.2) | 0.17 |
| Triglyceride, median (IQR), mg/dL | 143.5 (107.2–201.1) | 162.1 (115.1–245.3) | <0.01 |
| Cholesterol, median (IQR), mg/dL | 195.1 (165.3–234.5) | 193 (162–238.2) | 0.81 |
| High-density lipoprotein cholesterol, median (IQR), mg/dL | 54.1 (41–70.4) | 45.6 (37.9–61.5) | <0.01 |
| Low-density lipoprotein cholesterol, median (IQR), mg/dL | 107.5 (78.5–140.8) | 106.1 (80.4–141.9) | 0.89 |
| Renin-angiotensin-aldosterone system inhibitors, n (%) | 178 (76.7) | 207 (78.7) | 0.59 |
| New oral hypoglycemic agents, n (%) | 75 (32.3) | 88 (33.5) | 0.79 |
| Insulin therapy, n (%) | 158 (68.1) | 176 (66.9) | 0.92 |

Data are presented as the mean (standard) for continuous variables with symmetric distribution, median (25th-75th percentiles) for continuous variables with asymmetric distribution, or percentages for categorical variables.

**Abbreviations:** SD, standard deviation; IQR, interquartile range.

**Supplementary Table 3. Clinical characteristics of patients stratified by metabolically healthy status**

| Characteristics | Metabolically healthy (n=84） | Metabolically unhealthy (n=411） | *p* value |
| --- | --- | --- | --- |
| Age, mean (SD), y | 51 (9) | 51 (9) | 0.84 |
| Sex, Male, n (%) | 62 (73.8) | 284 (69.1) | 0.39 |
| Race, Tibetan, n(%) | 13 (15.5) | 49 (11.9) | 0.37 |
| Smoking, Never/Ex/Current, (n) | 50/8/26 | 219/67/125 | 0.27 |
| Body mass index, mean (SD), kg/m2 | 22 (3) | 25 (4) | <0.001 |
| Systolic blood pressure, mean (SD), mmHg | 122 (9) | 147 (23) | <0.001 |
| Diastolic blood pressure, mean (SD), mmHg | 78 (6) | 87 (13) | <0.001 |
| History of diabetic retinopathy, n (%) | 42 (50.0) | 220 (53.5) | 0.55 |
| Duration of diabetes, median (IQR), months | 84 (36–120) | 96 (36–132) | 0.27 |
| Hemoglobin A1c, median (IQR), % | 7 (5.9–8.1) | 7.4 (6.3–8.7) | 0.05 |
| Hemoglobin A1c, median (IQR), mmol/mol | 53 (41–65) | 57 (45–71) | 0.05 |
| Hemoglobin, mean (SD), g/L | 116.9 (24.5) | 119.7 (27.2) | 0.39 |
| Serum albumin, mean (SD), g/L | 33.9 (7.1) | 33.8 (7.8) | 0.98 |
| Fasting plasma glucose, median (IQR), mg/dL | 124 (93.2–160.2) | 133.2 (102.6–177.1) | 0.06 |
| Estimated glomerular filtration rate, median (IQR), mL/min/1.73 m^2^ | 67.5 (49.7–98.7) | 58 (40.3–87.8) | 0.01 |
| 24-h proteinuria, median (IQR), g/d | 3.8 (1.49–5.8) | 4.34 (2–7.63) | 0.13 |
| Hematuria, n (%) | 41 (48.8) | 168 (40.9) | 0.18 |
| Uric acid, mean (SD), mg/dL | 0.6 (0.1) | 0.6 (0.2) | 0.37 |
| Triglyceride, median (IQR), mg/dL | 112.5 (82.4–136) | 165.6 (119.6–238.3) | <0.001 |
| Cholesterol, median (IQR), mg/dL | 184.5 (163.8–211.3) | 197.6 (164.3–242.8) | 0.03 |
| High-density lipoprotein cholesterol, median (IQR), mg/dL | 56.5 (46.4–69.6) | 46.6 (37.9–63.8) | <0.001 |
| Low-density lipoprotein cholesterol, median (IQR), mg/dL | 104.8 (82.4–128) | 107.7 (78.5–144.6) | 0.37 |
| Renin-angiotensin-aldosterone system inhibitors, n (%) | 65 (77.4) | 320 (77.9) | 0.92 |
| New oral hypoglycemic agents, n (%) | 24 (28.6) | 139 (33.8) | 0.35 |
| Insulin therapy, n (%) | 56 (66.7) | 278 (67.6) | 0.99 |

Data are presented as the mean (standard) for continuous variables with symmetric distribution, median (25th-75th percentiles) for continuous variables with asymmetric distribution, or percentages for categorical variables.

**Abbreviations:** SD, standard deviation; IQR, interquartile range.

**Supplementary Table 4. Renal pathological characteristics of patients** **with non-obese and with obese**

| Characteristics | Non-obesity (n=232） | Obesity (n=263) | *p* value |
| --- | --- | --- | --- |
| Renal Pathology Society classification^†^, n (%) |  |  | 0.18 |
| I+IIa | 58 (25.0) | 62 (23.6) |  |
| IIb | 52 (22.4) | 42 (16.0) |  |
| III | 93 (40.1) | 114 (43.3) |  |
| IV | 29 (12.5) | 45 (17.1) |  |
| Global glomerulosclerosis, (%) | 25 (11.1–42.9) | 28.6 (12.5–48) | 0.13 |
| Segmental sclerosis, (%) | 0 (0–10.5) | 0 (0–10.8) | 0.98 |
| Presence of Kimmelstiel–Wilson nodule, n (%) | 94 (40.5) | 147 (55.9) | <0.01 |
| Presence of extracapillary hypercellularity, n (%) | 21 (9.1) | 16 (6.1) | 0.21 |
| Presence of microaneurysm, n (%) | 108 (46.6) | 107 (40.7) | 0.19 |
| Mesangial expansion |  |  | <0.01 |
| score 0 | 7 (3.0) | 6 (2.3) |  |
| score 1 | 116 (50.0) | 91 (34.6) |  |
| score 2 | 109 (47.0) | 166 (63.1) |  |
| Interstitial fibrosis and tubular atrophy^†^, n (%) |  |  | 0.09 |
| score 0 | 7 (3.0) | 14 (5.3) |  |
| score 1 | 127 (54.7) | 117 (44.5) |  |
| score 2 | 69 (29.7) | 99 (37.6) |  |
| score 3 | 29 (12.5) | 33 (12.5) |  |
| Interstitial inflammation^†^, n (%) | |  | 0.52 |
| score 0 | 6 (2.6) | 11 (4.2) |  |
| score 1 | 163 (70.3) | 188 (71.5) |  |
| score 2 | 63 (27.2) | 64 (24.3) |  |
| Arteriosclerosis^†^, n (%) |  |  | 0.84 |
| score 0 | 27 (11.6) | 35 (13.3) |  |
| score 1 | 109 (47.0) | 119 (45.2) |  |
| score 2 | 96 (41.4) | 109 (41.4) |  |
| Arteriolar hyalinosis^†^, n (%) | |  | 0.88 |
| score 0 | 27 (11.6) | 34 (12.9) |  |
| score 1 | 67 (28.9) | 72 (27.4) |  |
| score 2 | 138 (59.5) | 157 (59.7) |  |

Data are presented as percent for categorical variables. ^†^ Defined by Renal Pathology Society Diabetic Kidney Disease Classification.

**Supplementary Table 5. Renal pathological characteristics of patients stratified by metabolically healthy status**

| Characteristics | Metabolically healthy (n=84） | Metabolically unhealthy (n=411） | *p* value |
| --- | --- | --- | --- |
| Renal Pathology Society classification^†^, n (%) |  |  | 0.05 |
| I+IIa | 23 (27.4) | 97 (23.6) |  |
| IIb | 23 (27.4) | 71 (17.3) |  |
| III | 31 (36.9) | 176 (42.8) |  |
| IV | 7 (8.3) | 67 (16.3) |  |
| Global glomerulosclerosis, (%) | 22.2 (10–38.5) | 27.5 (12.5–50) | 0.06 |
| Segmental sclerosis, (%) | 0 (0–7.8) | 0 (0–11.1) | 0.75 |
| Presence of Kimmelstiel–Wilson nodule, n (%) | 33 (39.3) | 208 (50.6) | 0.06 |
| Presence of extracapillary hypercellularity, n (%) | 7 (8.3) | 30 (7.3) | 0.74 |
| Presence of microaneurysm, n (%) | 33 (39.3) | 182 (44.3) | 0.40 |
| Mesangial expansion |  |  | 0.37 |
| score 0 | 3 (3.6) | 10 (2.4) |  |
| score 1 | 40 (47.6) | 167 (40.6) |  |
| score 2 | 41 (48.8) | 234 (56.9) |  |
| Interstitial fibrosis and tubular atrophy^†^, n (%) |  |  | 0.04 |
| score 0 | 5 (6.0) | 16 (3.9) |  |
| score 1 | 51 (60.7) | 193 (47.0) |  |
| score 2 | 23 (27.4) | 145 (35.3) |  |
| score 3 | 5 (6.0) | 57 (13.9) |  |
| Interstitial inflammation^†^, n (%) | |  | 0.72 |
| score 0 | 4 (4.8) | 13 (3.2) |  |
| score 1 | 60 (71.4) | 291 (70.8) |  |
| score 2 | 20 (23.8) | 107 (26.0) |  |
| Arteriosclerosis^†^, n (%) |  |  | 0.02 |
| score 0 | 14 (16.7) | 48 (11.7) |  |
| score 1 | 47 (56.0) | 181 (44.0) |  |
| score 2 | 23 (27.4) | 182 (44.3) |  |
| Arteriolar hyalinosis^†^, n (%) | |  | 0.01 |
| score 0 | 16 (19.0) | 45 (10.9) |  |
| score 1 | 30 (35.7) | 109 (26.5) |  |
| score 2 | 38 (45.2) | 257 (62.5) |  |

Data are presented as percent for categorical variables. ^†^ Defined by Renal Pathology Society Diabetic Kidney Disease Classification.

**Supplementary Table 6. The *P* for interaction between obesity and metabolic status in type 2 diabetic patients with diabetic kidney disease.**

|  | Survival from end-stage kidney disease | |
| --- | --- | --- |
| Variables | Adjusted hazard ratio (95% Confidence Interval) ^#^ | *P* value _for interaction_ |
| Obesity | 2.09 (0.89–4.87) | 0.09 |
| Metabolically unhealthy status^†^ | 2.15 (1.19–3.87) | 0.01 |
| Obesity×Metabolically unhealthy status | 0.51 (0.21–1.23) | 0.14 |

^#^Adjusted for age, sex, baseline estimated glomerular filtration rate, 24-h proteinuria, uric acid, hemoglobin A1c, and serum albumin concentration, usage of renin-angiotensin-aldosterone system inhibitors, new oral hypoglycemic agent, and smoking plus pathological parameters (Renal Pathology Society glomerular classifications, interstitial fibrosis and tubular atrophy, interstitial inflammation, arteriosclerosis, arteriolar hyalinosis, Kimmelstiel–Wilson nodule, and mesangial expansion). ^†^ Comparison to metabolically healthy status.

**Supplementary Table 7. Univariate and multivariate analyses for ESKD in patients with type 2 diabetes and diabetic kidney disease after excluding the first one year of follow-up.**

| Variables | Univariate Models hazard ratio (95% confidence interval) | *P* value | Multivariable model 1^a^ adjusted hazard ratio (95% confidence interval) | *P* value | Multivariable model 2^b^ adjusted hazard ratio (95% confidence interval) | *P* value | Multivariable model 3^c^ adjusted hazard ratio (95% confidence interval) | *P* value |
| --- | --- | --- | --- | --- | --- | --- | --- | --- |
| Metabolic phenotype |  |  |  |  |  |  |  |  |
| MHNO | 1 (reference) |  | 1 (reference) |  | 1 (reference) |  | 1 (reference) |  |
| MHO | 1.16 (1.00–2.58) | 0.05 | 1.52 (0.65–3.56) | 0.34 | 2.64 (1.11–6.31) | 0.03 | 1.69 (0.67–4.28) | 0.27 |
| MUNO | 2.07 (1.19–3.59) | 0.01 | 1.9 (1.07–3.39) | 0.03 | 2.29 (1.28–4.12) | 0.01 | 2.32 (1.27–4.24) | 0.01 |
| MUO | 2.09 (1.22–3.57) | 0.01 | 2.01 (1.14–3.52) | 0.02 | 3.03 (1.69–5.45) | <0.001 | 2.45 (1.32–4.52) | <0.01 |
| Obesity | 1.11 (0.85–1.45) | 0.44 | 1.18 (0.89–1.55) | 0.25 | 1.54 (0.94–2.07) | 0.07 | 1.19 (0.86–1.65) | 0.28 |
| Metabolically unhealthy status^†^ | 1.97 (1.3–2.98) | <0.01 | 1.7 (1.1–2.65) | 0.02 | 1.98 (1.26–3.12) | <0.01 | 1.99 (1.25–3.15) | <0.01 |
| Number of metabolic abnormality components | 1.25 (1.1–1.42) | <0.001 | 1.19 (1.03–1.37) | 0.02 | 1.21 (1.05–1.41) | 0.01 | 1.25 (1.08–1.45) | <0.01 |

^a^ Adjusted for age, sex, baseline estimated glomerular filtration rate, 24-h proteinuria, uric acid, hemoglobin A1c, and serum albumin concentration. ^b^ Adjusted for the parameters in multivariable model a plus hemoglobin A1c, hemoglobin, and serum albumin concentration, usage of renin-angiotensin-aldosterone system inhibitors, new oral hypoglycemic agent, and smoking. ^c^ Adjusted for the parameters in multivariable model a plus pathological parameters (Renal Pathology Society glomerular classifications, interstitial fibrosis and tubular atrophy, interstitial inflammation, arteriosclerosis, arteriolar hyalinosis, Kimmelstiel–Wilson nodule, and mesangial expansion). ^†^ Comparison to metabolically healthy status.

**Abbreviations:** MHNO, metabolically healthy non-obesity; MHO, metabolically healthy obesity; MUNO, metabolically unhealthy non-obesity; MUO, metabolically unhealthy obesity.

**Supplementary Table 8. Univariate and multivariate analyses for ESKD in patients with type 2 diabetes and diabetic kidney disease after excluding the first one year of follow-up.**

| Variables | Univariate Models hazard ratio (95% confidence interval) | *P* value | Multivariable model 1^a^ adjusted hazard ratio (95% confidence interval) | *P* value | Multivariable model 2^b^ adjusted hazard ratio (95% confidence interval) | *P* value | Multivariable model 3^c^ adjusted hazard ratio (95% confidence interval) | *P* value |
| --- | --- | --- | --- | --- | --- | --- | --- | --- |
| Metabolic phenotype | |  |  |  |  |  |  |  |
| MHNO | 1 (reference) |  | 1 (reference) |  | 1 (reference) |  | 1 (reference) |  |
| MHO | 1.56 (0.8–3.05) | 0.2 | 1.65 (0.79–3.44) | 0.18 | 2.72 (1.29–5.76) | 0.01 | 1.77 (0.79–3.98) | 0.16 |
| MUNO | 1.93 (1.18–3.16) | 0.01 | 1.54 (0.92–2.57) | 0.1 | 1.83 (1.09–3.07) | 0.02 | 1.83 (1.07–3.13) | 0.03 |
| MUO | 1.89 (1.18–3.04) | 0.01 | 1.65 (1.01–2.69) | 0.05 | 2.46 (1.46–4.13) | <0.001 | 1.97 (1.15–3.4) | 0.01 |
| Obesity | 1.14 (0.88–1.48) | 0.33 | 1.19 (0.9–1.56) | 0.22 | 1.57 (0.97–2.10) | 0.06 | 1.22 (0.88–1.68) | 0.22 |
| Metabolically unhealthy status^†^ | 1.62 (1.13–2.32) | 0.01 | 1.36 (0.93–2.01) | 0.12 | 1.6 (1.07–2.38) | 0.02 | 1.56 (1.04–2.35) | 0.03 |

^a^ Adjusted for age, sex, baseline estimated glomerular filtration rate, 24-h proteinuria, uric acid, hemoglobin A1c, and serum albumin concentration. ^b^ Adjusted for the parameters in multivariable model a plus hemoglobin A1c, hemoglobin, and serum albumin concentration, usage of renin-angiotensin-aldosterone system inhibitors, new oral hypoglycemic agent, and smoking. ^c^ Adjusted for the parameters in multivariable model a plus pathological parameters (Renal Pathology Society glomerular classifications, interstitial fibrosis and tubular atrophy, interstitial inflammation, arteriosclerosis, arteriolar hyalinosis, Kimmelstiel–Wilson nodule, and mesangial expansion). ^†^ Comparison to metabolically healthy status.

**Abbreviations:** MHNO, metabolically healthy non-obesity; MHO, metabolically healthy obesity; MUNO, metabolically unhealthy non-obesity; MUO, metabolically unhealthy obesity.
